# Development of Superparamagnetic Nanoparticles Coated with Polyacrylic Acid and Aluminum Hydroxide as an Efficient Contrast Agent for Multimodal Imaging

**DOI:** 10.3390/nano9111626

**Published:** 2019-11-15

**Authors:** Manuel Antonio González-Gómez, Sarah Belderbos, Susana Yañez-Vilar, Yolanda Piñeiro, Frederik Cleeren, Guy Bormans, Christophe M. Deroose, Willy Gsell, Uwe Himmelreich, José Rivas

**Affiliations:** 1 Applied Physics Department, NANOMAG Laboratory, Universidade de Santiago de Compostela, 15782 Santiago de Compostela, Spain; susana.yanez@usc.es (S.Y.-V.); y.pineiro.redondo@usc.es (Y.P.); jose.rivas@usc.es (J.R.); 2 Biomedical MRI, Department of Imaging and Pathology, KU Leuven, O&N I, Herestraat 49—Box 505, 3000 Leuven, Belgium; willy.gsell@kuleuven.be (W.G.); uwe.himmelreich@kuleuven.be (U.H.); 3 Molecular Small Animal Imaging Center (MoSAIC), KU Leuven, O&N I, Herestraat 49—Box 505, 3000 Leuven, Belgium; 4 Radiopharmaceutical Research, Department of Pharmaceutical and Pharmacological Sciences, KU Leuven, O&NII Herestraat 49—Box 821, 3000 Leuven, Belgium; frederik.cleeren@kuleuven.be (F.C.); guy.bormans@kuleuven.be (G.B.); 5 Nuclear Medicine and Molecular Imaging, Department of Imaging and Pathology, KU Leuven/UZ Leuven, Herestraat 49—Box 7003 59, 3000 Leuven, Belgium; christophe.deroose@uzleuven.be

**Keywords:** magnetite (Fe_3_O_4_), superparamagnetic nanoparticles (SPMNPs), aluminum hydroxide (Al(OH)_3_), multimodal imaging, MRI, PET, biomedical applications

## Abstract

Early diagnosis of disease and follow-up of therapy is of vital importance for appropriate patient management since it allows rapid treatment, thereby reducing mortality and improving health and quality of life with lower expenditure for health care systems. New approaches include nanomedicine-based diagnosis combined with therapy. Nanoparticles (NPs), as contrast agents for in vivo diagnosis, have the advantage of combining several imaging agents that are visible using different modalities, thereby achieving high spatial resolution, high sensitivity, high specificity, morphological, and functional information. In this work, we present the development of aluminum hydroxide nanostructures embedded with polyacrylic acid (PAA) coated iron oxide superparamagnetic nanoparticles, Fe_3_O_4_@Al(OH)_3_, synthesized by a two-step co-precipitation and forced hydrolysis method, their physicochemical characterization and first biomedical studies as dual magnetic resonance imaging (MRI)/positron emission tomography (PET) contrast agents for cell imaging. The so-prepared NPs are size-controlled, with diameters below 250 nm, completely and homogeneously coated with an Al(OH)_3_ phase over the magnetite cores, superparamagnetic with high saturation magnetization value (Ms = 63 emu/g-Fe_3_O_4_), and porous at the surface with a chemical affinity for fluoride ion adsorption. The suitability as MRI and PET contrast agents was tested showing high transversal relaxivity (r_2_) (83.6 mM^−1^ s^−1^) and rapid uptake of ^18^F-labeled fluoride ions as a PET tracer. The loading stability with ^18^F-fluoride was tested in longitudinal experiments using water, buffer, and cell culture media. Even though the stability of the ^18^F-label varied, it remained stable under all conditions. A first in vivo experiment indicates the suitability of Fe_3_O_4_@Al(OH)_3_ nanoparticles as a dual contrast agent for sensitive short-term (PET) and high-resolution long-term imaging (MRI).

## 1. Introduction

Advances in physics and engineering have allowed the development of non-invasive imaging techniques that gave rise to a striking medical revolution in which detailed physiology, pathology, and functionality of the human body could be revealed with detailed precision non-invasively. Among others, X-ray, magnetic resonance imaging (MRI), and positron emission tomography (PET), have settled in as the core technologies that constitute a milestone in medical practice [1,2].

In the quest for increasing the potential of these imaging techniques, nuclides used as contrast agents like Gd, ^18^F, ^68^Ga [3] have become a crucial tool for revealing subtle differences between tissues allowing for enhanced accuracy of diagnosis and assessment of the effectiveness of therapies in the last decades. 

With the appearance of dual imaging modalities (PET/MRI, PET/computed tomography (CT), and single-photon emission computed tomography (SPECT)/CT), there is not only a potential for further improved diagnosis but also a continuous need for developing more versatile and less harmful contrast agents, capable to provide multimodal contrast in a single formulation, robust effectiveness under minimal dosages, negligible secondary effects, and stable performance for long-term follow-up using functional diagnosis procedures [4,5,6,7,8].

Compared to the conventional use of small molecules as contrast agents for in vivo and in vitro imaging, nanomaterials offer significant advantages and are providing important contributions to the field of diagnostic and non-invasive molecular imaging, which includes the generation of stronger and long-lasting contrast and the combination with therapy delivery in theranostic agents [9,10]. 

Recently, magnetite iron oxide nanoparticles (Fe_3_O_4_ NPs) have attracted considerable interest as negative MRI contrast agents due to their excellent magnetic properties, low toxicity, biocompatibility, and biodegradability compared to the other magnetic NPs (MnFe_2_O_4_, ZnFe_2_O_4_, CoFe_2_O_4_, etc.) [11]. For this application, Fe_3_O_4_ nanoparticles with a narrow size distribution, superparamagnetic behavior, and specific surface modification are desired [12,13]. This surface modification can increase the properties of biocompatibility and avoid the agglomeration of the bare particles due to the magnetostatics interaction [14]. Many materials and coating approaches have been utilized for modifying the surface of these nanoparticles. Polymers are the most widely used coating materials and can be classified as hydrophilic or amphiphilic, neutral or charged, and homopolymers or copolymers. Amongst them, coating with polyethylene glycol (PEG), Dextran, or polyacrylic acid [14,15,16] has been widely used. Also, to avoid desorption of the polymeric coating by heating or dilution, one or more functional groups, such as carbonate or phosphonate, are necessary to bind with the NPs. Therefore, the use of an inorganic shell material that introduces stability, functionality, and water-solubility is desirable.

Their versatile performance has already given rise and excellent T_2_/T_2_* contrast for MRI providing high-resolution images for in vivo cell tracking [17,18] and clinically tested and approved formulations for commercialization as contrast agents for MRI (Feridex; Resovist, Ferumoxtran-10) [19], or as therapeutic agents in magnetic hyperthermia therapies for brain tumor treatment (NanoTherm) [14,20].

However, one disadvantage of the T_2_/T_2_* MR contrast generated by superparamagnetic (SPM) iron oxide NPs is that this hypointense signal change (darkening) is not suitable for imaging against a hypointense background like it is the case in MR images of the lungs or parts of the abdomen. In addition, MRI lacks the high sensitivity of imaging techniques like PET. Therefore, contrast agents that generate dual contrast for MRI and PET can help to overcome the limitations of individual imaging techniques [21,22]. SPM nanoparticles, with an engineered composition, offer a platform to incorporate different components into one single entity. Specifically, dual PET/MRI contrast agents could be obtained combining a magnetite core (Fe_3_O_4_) for MRI contrast embedded in an aluminum hydroxide shell (Al(OH)_3_), which can be labeled with ^18^F-fluoride ions as a PET tracer [23,24]. 

In the present work, we have developed hydrophilic and colloidally stable Fe_3_O_4_@Al(OH)_3_ nanostructures, with almost spherical homogeneous size-morphology, to be used as dual MRI/PET contrast agents, by a relatively simple and controllable chemistry method [25,26,27,28]. Magnetite cores with good crystalline quality, SPM response, T_2_ contrast, and controlled size, were prepared by a co-precipitation method, based on mild starting reagents and low temperatures, suitable for scaling up purposes. Colloidal stability [29] was ensured by adding a layer of polyacrylic acid (PAA), which was selected for its strong coordination of carboxylate groups with iron cations on the magnetite surface and their high degree of dispersibility in water [30,31]. Al(OH)_3_ external coating shell was incorporated by a forced hydrolysis procedure, providing the nanostructure with a porous and positively charged surface with an affinity for fluoride ions to be used in PET imaging. This approach was tested for in vitro mesenchymal stem cell (MSC) labeling, and cytotoxicity assessment of the NPs for future cell tracking applications. The visualization of cells was demonstrated in vivo in a preliminary PET/MRI experiment. 

## 2. Materials and Methods

### 2.1. Chemicals and Synthesis

#### 2.1.1. Chemicals

Chemicals used for this study were, aluminum nitrate nonahydrate (Al(NO_3_)_3_·9H_2_O, 98%), aluminum sulfate hexadecahydrate (Al_2_(SO_4_)_3_·16H2O, 98%), iron(III) chloride hexahydrate (FeCl_3_·6H_2_O, 99%), iron(II) sulfate heptahydrate (FeSO_4_·7H_2_O, 99%), hydrochloric acid (HCl, 37%), nitric acid (HNO_3_, 65%), ammonium hydroxide (NH_4_OH, 28%), sodium fluoride (NaF, ≥99%), polyacrylic acid ((C_3_H_4_O_2_)_n_ average Mw 1800), urea (CON_2_H_4_, 98%) and agar all obtained from Sigma-Aldrich (St. Louis, MO, USA). Total Ionic Strength Adjustment Buffer solution (TISAB III), phosphate-buffered saline (PBS), TrypLE, high-glucose Dulbecco’s Modified Eagle’s Medium (DMEM) containing GlutaMax, fetal bovine serum (FBS), and penicillin-streptomycin were obtained from Gibco-Invitrogen (Gibco-Invitrogen™-Fisher, Carlsbad, CA, USA), Milli-Q (Millipore®, Burlington, MA, USA) deionized water was used in all the experiments.

#### 2.1.2. Synthesis of Fe_3_O_4_ NPs Functionalized with PAA

MNPs were obtained by co-precipitation, following Massart’s method [32] with some modifications. In a typical synthesis, FeCl_3_•6H_2_O (45 mmol) and FeSO_4_•7H_2_O (30 mmol) were dissolved in 100 mL of 10 mM HCl aqueous solution with mechanically stirring. The mixture was heated at 60 °C, then NH_4_OH (770 mmol) and PAA (1.11 mmol) were added, and the reaction was carried out for 1 h. After that, the obtained magnetic nanoparticles were acidified up to pH 5 with the incorporation of the HCl solution at 9%. Magnetite NPs were separated from the reaction medium by a magnetic field and washed several times (6×) with Milli-Q water. Finally, Fe_3_O_4_@PAA NPs were re-dispersed in Milli-Q water. The total solid content was determined by thermogravimetric analysis (TGA): W_mag_ = 5.5% by weight. 

#### 2.1.3. Synthesis of Functionalized Fe_3_O_4_ NPs with PAA Coated Then by a Layer of Al(OH)_3_

Al(OH)_3_ coating on the Fe_3_O_4_@PAA NPs was prepared by a forced chemical hydrolysis method as reported by Roh et al. [33] with some alterations. Briefly, 200 mg magnetite coated with PAA nanoparticles were added in 250 mL Milli-Q water, after sonication for 15 min to form a homogenous solution, Al(NO_3_)_3_·9H_2_O (11.6 mmol), Al_2_(SO_4_)_3_·16H_2_O (1.92 mmol), and urea (0.5 mol) were added. The reactant solution was added in an oil bath of 98 °C, with mechanical stirring for 1.5 h. The obtained nanomaterial was magnetically separated, washed, and re-dispersed in Milli-Q water. The total solids content was determined by TGA: W_mag_ = 2.46% by weight.

### 2.2. Physicochemical Characterization

#### 2.2.1. XRD- Structural Characterization

The characterization of the crystalline phases was performed by X-ray diffraction (XRD) with powder samples using a Philips PW1710 diffractometer (Panalytical, Brighton, UK) with a Cu Kα radiation source, λ = 1.54186Å. Measurements were collected in the 2θ angle range between 10° and 80° with steps of 0.02° and 10 s/step.

#### 2.2.2. Microscopy Morphological Characterization

Morphology of the materials was characterized by scanning electron microscopy (SEM) using a Zeiss FE-SEM ULTRA Plus (5 kV) microscope (Zeiss, Oberkochen, Germany). Transmission electron microscopy (TEM) was realized with High-Resolution Transmission Electron Microscopy LIBRA 200FE with field-emission gun and OMEGA Energy Filter (Carl Zeiss NTS GmbH, Oberkochen, Germany). The TEM measurements were performed at 200 kV and the High-Angle Annular Dark Field (HAADF). Scanning transmission electron microscopy (STEM) images were performed using a HAADF detector in STEM mode, and Z-contrast differences were visualized. The dispersive energy analyses were performed with an Oxford energy dispersive X-ray (EDX) detector. 

#### 2.2.3. Surface Chemistry Characterization

Fourier transform infrared (FTIR) spectra of the surface functional groups of the nanostructures were recorded with a Thermo Nicolet Nexus spectrometer (Thermo Fisher Scientific, Madrid, Spain) using the attenuated total reflectance (ATR) method from 4000 to 400 cm^−1^.

#### 2.2.4. Compositional Characterization

Iron content in the nanocomposite samples was determined by flame atomic absorption spectroscopy (FAAS) performed with an Atomic Absorption Spectrometer (Perkin Elmer 3110, Perkin, Waltham, MA, USA).

The composition of the samples was analyzed with a TGA Perkin Elmer model 7 (Perkin, Waltham, MA, USA).

The adsorption percentage of non-radioactive fluoride was determined using a pH & Ion-Meter BASIC 20+ (CRISON, Barcelona, Spain).

#### 2.2.5. Magnetic Characterization

Direct current (DC) magnetization curves of dried samples were measured using a vibrating sample magnetometer (VSM) (DMS, Lowell, MA, USA). In such a device, the measurement of magnetic hysteresis loops at room temperature was carried out under external magnetic fields from −10 to 10 kOe.

#### 2.2.6. Preliminary Study of Non-Radioactive ^19^F Adsorption

The adsorption capacity of Fe_3_O_4_@Al(OH)_3_ NPs of fluoride ions (non-radioactive ^19^F) was assessed with the help of an ion-selective electrode (ISE) method, using as working and reference electrodes fluoride and silver, respectively. For this purpose, from an initially prepared solution of NaF (1 g/L), a set of 25 solutions were prepared by mixing 10 mL of aliquots with defined concentrations (from 20 to 500 mg/L) with 10 mg of Fe_3_O_4_@Al(OH)_3_ NPs at pH 6.5. The mixtures were incubated at 25 °C during 10 h at 250 rpm with orbital stirring to facilitate the adsorption of fluoride ions on the aluminum hydroxide shell. Afterward, the magnetic nanostructures (Fe_3_O_4_@Al(OH)_3_) were separated from the solution with a neodymium magnet (NdFeB), and the supernatants were collected for ISE analysis. As in a typical experiment, 5 mL of supernatant was mixed with 25 mL of Total Ionic Strength Adjustment Buffer solution (TISAB III) to avoid interference of Al(III) and Fe(III) with the electrodes. In this way, metal ions form stable complexes by reacting with 1,2-Cyclohexylenedinitrilotetraacetic acid (buffer component), and fluoride ions are free and available for their concentration analysis based on pH and ion-meter with an ISE of fluoride and silver.

#### 2.2.7. Labeling of Fe_3_O_4_@Al(OH)_3_ Nanoparticles with ^18^F-Sodium Fluoride

Fifty µL containing 5 to 7 MBq [^18^F]NaF were added to tubes containing 1, 2, 5, or 10 mg of Fe_3_O_4_@Al(OH)_3_ NPs in 500 µL of water. Tubes were continuously shaken for 15 min at room temperature. This was followed by separation of the nanoparticles by either centrifugation (3000 rpm for 20 min) or by magnetic separation using a permanent magnet and then were washed three times with water. The radioactivity of the particles and supernatants were measured after each washing step using a gamma counter. All measurements were performed in triplicates.

#### 2.2.8. [^18^F]NaF Production

Fluorine-18 was produced by irradiation of H_2_^18^O with 18-MeV protons using a cyclotron (IBA Cyclone 18/9, IBA, Louvain-la-Neuve, Belgium). [^18^F]F^−^ was separated from [^18^O]H_2_O by trapping on a Sep-Pak Light Accell plus QMA anion exchange cartridge (Cl^−^ form; Waters, Zellik, Belgium). The cartridge was washed with water (3 mL, HPCE grade; Sigma-Aldrich, Overijse, Belgium) and [^18^F]F^−^ was eluted from the cartridge with an aqueous solution of sodium chloride 0.9% (Mini-Plasco, solution for injection, B. Braun, Diegem, Belgium) to obtain a concentration of 0.8–3.0 GBq/mL at the end of synthesis.

#### 2.2.9. Radiolabeling of Fe_3_O_4_@Al(OH)_3_ NPs

To evaluate the time course of radiolabeling, 45 µL of Fe_3_O_4_@Al(OH)_3_ NPs containing 60 µg of Fe were incubated with 5–30 MBq [^18^F]NaF (5–30 µL) while shaking. Two, five, and 10 min after labeling, 2 µL samples were taken and blotted on instant thin layer chromatography (iTLC) papers impregnated with silica gel (iTLC-SG papers; Varian, Diegem, Belgium). The papers were developed in an elution chamber using NaCl 0.9% as the mobile phase. The read-out was performed using a 2480 Wizard^2^ Automatic Gamma Counter (20 s protocol; PerkinElmer, Waltham, MA, USA) after splitting the papers into two equal halves representing the unbound and bound radiotracer. In addition, autoradiography was performed using phosphor screens (Perkin Elmer) in standard film cassettes. The screens were removed from the cassettes after a five-minute exposure and were scanned immediately at 300 dpi resolution using a Cyclone Plus System (Perkin Elmer). Images were analyzed using the manufactures’ Optiquant software (version 5; Perkin Elmer).

For further in vitro and in vivo experiment, NPs are labeled with [^18^F]NaF for ten minutes in Milli-Q water. Afterward, they are centrifuged for 20 min at 4000 rpm and resuspended in the media of choice for further application.

#### 2.2.10. Stability of the Radiolabeling

The adsorption stability of [^18^F]F^−^ to the Fe_3_O_4_@Al(OH)_3_ NPs was evaluated after centrifugation (20 min at 4000 rpm) of the NPs (11.85 µg iron, labeled with 5 MBq [^18^F]NaF). The supernatant was removed and the labeled NPs were resuspended in 500 µL of one of the following media: (1) Milli-Q water, (2) saline (sodium chloride 0.9%), (3) PBS, (4) TrypLE, (5) high-glucose DMEM containing GlutaMax and 15% FBS, (6) 50% DMEM + GlutaMax and 50% FBS, or (7) FBS. All components used in conditions (3)–(7) were obtained from Gibco (ThermoFisher Scientific, Erembodegem, Belgium). Afterward, NPs were exposed to the media for 30 min, 1 h, 2 h, and 4 h at 37 °C. At the respective time points, 2 µL samples were taken for iTLC.

In a second stability experiment, NPs were resuspended in either (1) Milli-Q, (2) high-glucose DMEM containing GlutaMax, and 15% FBS, (3) 50% DMEM + GlutaMax, and 50% FBS or (4) FBS. NPs were incubated for 15 min and centrifuged for 20 min at 4000 rpm. After the centrifugation step, the supernatant was removed, and 2 µL of the pellet consisting of NPs and 2 µL of the supernatant were collected for iTLC measurements. Remaining NPs were then resuspended in a fresh medium. All steps were repeated four times to determine.

iTLC was performed as described above. After splitting the iTLC papers into the bottom and top halves (representing the nanoparticle and medium fraction), the radioactivity present on each half was measured using a 20 s protocol on the gamma-counter. The percentage of the unbound and NP-bound fraction of [^18^F]F^−^ was calculated based on the total radioactivity present in the mixture.

#### 2.2.11. Dual PET/MRI Scanning

PET/MR images were simultaneously acquired using a Bruker BioSpec 70/30 small animal MRI scanner equipped with a PET insert (3 rings composed of eight monolithic lutetium-yttium oxyorthosilicate (LYSO) crystals coupled to SiPMs; Bruker Biospin, Ettlingen, Germany) [34]. The MR scanner with a horizontal bore of 30 cm was equipped with actively shielded gradients (200 mT m^−1^). A quadrature radio-frequency resonator (transmit/receive; inner diameter 7.2 cm or 8.6 cm for in vivo and in vitro scans, respectively; Bruker Biospin) was used for image acquisition. A one-hour static PET scan and the following parametric MRI maps were simultaneously acquired in vitro: T_2_* (multigradient-echo (MGE) with e echo time (TE) = 4–57.3 ms with 4.1 ms increments, repetition time (TR) = 7000 ms, 4 averages, matrix = 256 × 256), T_2_ (multi-slice multi-echo (MSME), TE = 14–280 ms with 14 ms increments, TR = 5000 ms, 2 averages, matrix = 258 × 258) and T_1_ (spin-echo sequence with slice selective inversion recovery, TE = 7.45 ms, recovery time = 10,000 ms, inversion times (TI) = 50–5500 ms 500, matrix = 128 × 128) maps. All in vitro scans have a field of view (FOV) 60 × 60 mm and contain five slices of 1 mm thickness with a 1 mm gap between slices.

### 2.3. Biological Characterization

#### 2.3.1. Cell Labelling Using Radiolabelled Fe_3_O_4_@Al(OH)_3_ NPs

Mouse mesenchymal stem cells (mMSCs) transduced with a lentiviral vector encoding for firefly luciferase and the enhanced green fluorescent protein were cultured in high-glucose DMEM containing GlutaMax supplemented with 15% FBS and 1% penicillin-streptomycin (Gibco). Cells were grown in a humidified incubator at 37 °C, 21% O_2_, and 5% CO_2_ (Binder, Tuttlingen, Germany).

One day after mMSCs plating, cells were labeled with radiolabeled (RL) NPs (containing 0.38 mM iron, diluted in saline) for 1 h. Next, the NP-containing saline was removed, cells were washed with saline, and 0.5 mL TrypLE was added (approximately 5 min exposure) to detach the mMSCs from the surface. Total cell count was performed using a Neubauer cell counting chamber (Hirschmann, Eberstadt, Germany). Details on NP iron content and the amount of radioactivity are provided below.

#### 2.3.2. Iron Content Measurement in Cell Labelling

To assess cell labeling stability, 100 µL of the sample (NPs or NP-labeled cells) was digested with 100 µL of HNO_3_. Samples were diluted using distilled water to reach an exact volume of 5 mL. The iron concentration was measured by inductively coupled plasma optical emission spectroscopy (ICP-OES; using a Varian 720ES; Varian). The intensity of the emission at 238.204 nm was used for iron quantification.

#### 2.3.3. In Vitro PET/MR of RL Fe_3_O_4_@Al(OH)_3_ NPs and RL NP-Labelled mMSCs

Fe_3_O_4_@Al(OH)_3_ NPs containing 4.3 µg of iron were labeled with 10 MBq [^18^F]NaF, while 10^6^ mMSC plated the day before where labeled with NPs containing 21.4 µg of iron in 1 mL of saline (end concentration of iron on cells = 0.38 mM) and radiolabeled with 30 MBq [^18^F]NaF. For imaging purposes, serial dilutions of these NPs and cells were resuspended in equal amounts of saline and 2% agar (microbiology grade; Sigma-Aldrich; diluted in distilled water) in 200 µL Eppendorf tubes. The following controls were used: (1) equal amounts of saline and 2% agar, and (2) 5 × 10^4^ unlabeled mMSCs in saline and agar. The radioactivity present in the samples was measured using a dose calibrator (CRC-15 PET, Capintec Inc., Ramsey, NJ, USA). The tubes were then placed into a cylindrical in-house made Teflon holder (outer diameter = 7 cm, inner diameter = 3.5 cm) filled with 2% liquid agar. For more details on this phantom, see Trekker et al. [35]. After agar solidification, the phantom was scanned using PET/MRI. Three MRI measurements were performed per phantom.

#### 2.3.4. Image Analysis

##### MRI

All parametric maps, except T_2_* maps, were generated using the Paravision software (Bruker Biospin, version 6.0.1). Due to the need for Eddy’s current correction, the following procedure was used to generate the T_2_* maps: for each slice, the images for the TE 2–14 were co-registered to the image of the first TE using the TurboReg plugin [36]. Afterward, parametric maps (exponential fitting) were generated using an in-house written Python script. To quantify the T_2_*, T_2_, and T_1_ values of the NPs/NP-labeled mMSCs, circular regions of interest were drawn within the images of Eppendorf tubes on one slice of the scan. 

##### PET

The manufacturer’s Albira software (Bruker Biospin) was used to reconstruct the acquired PET scans. One-hour static scans were reconstructed using a maximum likelihood estimation method (MLEM; parameters = 12 iterations, 0.5 mm isotropic resolution, decay/scatter/random correction). Reconstructed PET images were analyzed in PMOD version 3.9 (PMOD Technologies, Zürich, Switzerland). 

The PET scan was overlaid with the MR images using the PMOD FuseIT tool. Volumes of interest were drawn around the contours of the Eppendorf tubes to quantify the radioactivity present in each tube.

#### 2.3.5. Cell Proliferation and Survival

Putative toxic effects due to the labeling procedure were performed by Trypan Blue exclusion as a measure of cell viability/immediate cell death rates after overnight exposure (12 h) of MSCs to different concentrations of Fe_3_O_4_@Al(OH)_3_ NPs. Long-term effects of the cell labeling with the iron oxide contrast agents were studied after washing the cells and continued incubation of the cell lines in iron-free medium and compared with unlabeled controls. Inhibition of proliferation was used as an additional measure for potentially toxic effects. Hereby, population doubling times were determined using t × ln (2)/ln (A/A0), where t is the time between two cell counts, A is the number of cells at the end of the incubation, and A0 is the initial number of cells [37]. 

#### 2.3.6. Statistical Analysis

All statistical analyses were performed in GraphPad Prism version 5.0.4 (GraphPad Software, San Diego, CA, USA). Linear regressions of the relaxation rates (R_x_) versus the iron concentration present in the samples were used for determining the NPs/mMSCs relaxivities and relaxation rates. Correlations between the number of NPs/cells and radioactivity were calculated using a Pearson’s correlation test. Comparisons between the groups were analyzed using a one-way ANOVA with Bonferroni correction. Comparisons between the groups at different time points were studied using a two-way repeated-measures ANOVA or mixed-effect analysis in case of missing data with Bonferroni correction for multiple comparisons. Differences were considered statistically significant when *p* < 0.05. In all the figures, data are represented as mean ± standard deviation (SD).

## 3. Results and Discussion

### 3.1. X-ray Diffraction (XRD)

**Figure 1:**
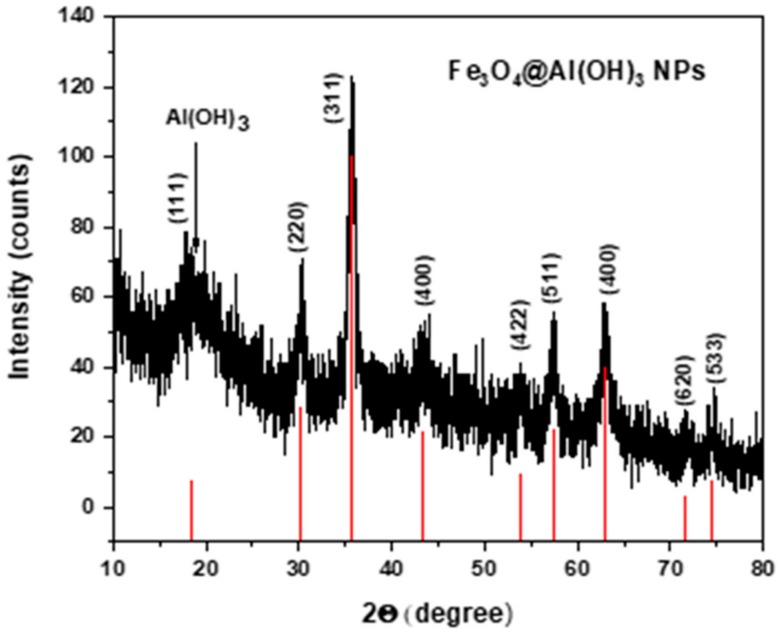
X-ray diffraction (XRD) pattern of the core/shell structure of magnetite/aluminum hydroxide nanoparticles, compared to the XRD pattern of magnetite from the JCPDS 19-0629 data base.

In Figure 1, the experimental pattern of the NPs is presented together with the theoretical diffraction peaks of magnetite, (JCPDS card No. 19-0629) [38] showing that both, the location and relative intensity of Fe_3_O_4_@Al(OH)_3_ NPs coincide with the main theoretical 111, 220, 311, 400, 422, 511, 440 magnetite reflections. This confirms that magnetite is the iron oxide crystalline phase present in the sample. In addition, located at 20°, a broadband which is overlapping with the (110) reflection of Fe_3_O_4_ can be observed corresponding to the Al(OH)_3_ shell that can be ascribed to the amorphous boehmite phase (JCPDS card No. 20-0011) [39,40].

### 3.2. Fourier-Transform Infrared Spectroscopy (FTIR) Spectroscopy

**Figure 2:**
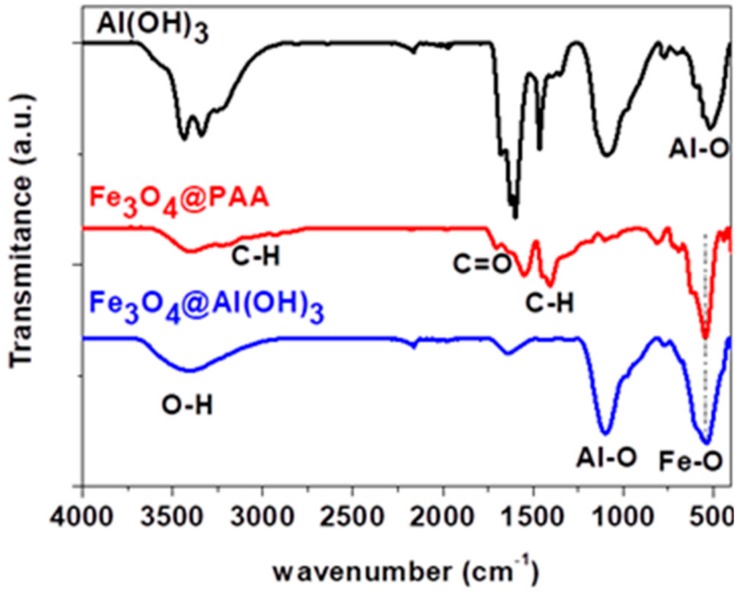
Fourier transform infrared (FTIR) spectra of aluminum hydroxide nanoparticles (NPs), magnetite coated with polyacrylic acid NPs and polyacrylic acid magnetic nanoparticles coated with aluminum hydroxide.

Figure 2 shows the FT-IR spectra of Al(OH)_3_, Fe_3_O_4_@PAA, and Fe_3_O_4_@Al(OH)_3_ nanoparticles. The peak observed around 550 cm^−1^ is characteristic of Fe-O vibrations [41], whereas the peaks at 1407, 2851, and 2923 cm^−1^ are related to -CH_2_^−^ groups, proving the presence of PAA in the sample. Furthermore, a peak at 1701 cm^−1^ is also observed, related to the carboxyl group [42]. All these results reveal that the surface of Fe_3_O_4_ NPs is successfully modified with PAA in the Fe_3_O_4_@PAA nanoparticles. The Al(OH)_3_ nanoparticles exhibit peaks at 510 and 1082 cm^−1^ that correspond to the Al-O bonds, which are also observed in the Fe_3_O_4_@Al(OH)_3_ NPs. A large band around 3400 cm^−1^ is also observed, due to the -OH groups adsorbed on the nanoparticle surface.

### 3.3. Magnetic Characterization

**Figure 3:**
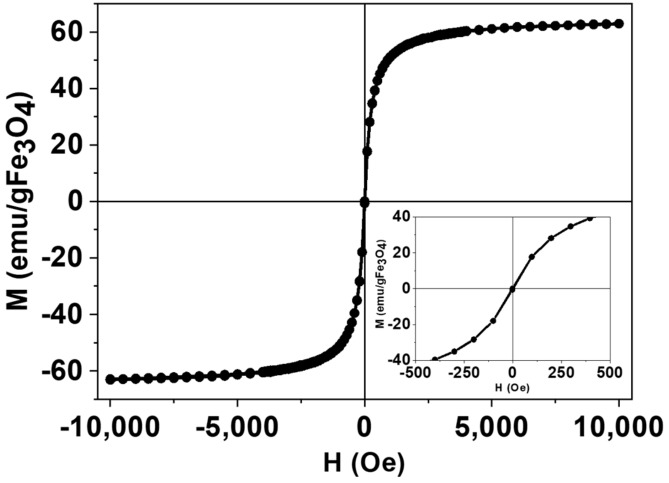
Magnetic hysteresis loop of a representative sample of Fe_3_O_4_@Al(OH)_3_ NPs, normalized to the magnetite content and showing nearly superparamagnetic (SPM) behavior, with negligible values of coercivity and remanence.

Figure 3 show the magnetization curves versus the applied magnetic field from −10 kOe to +10 kOe, measured on dried samples of Fe_3_O_4_@Al(OH)_3_ at room temperature with a Vibrating Sample Magnetometer (VSM). Magnetization data were normalized to the amount of magnetite mass (Wmag) for each sample (determined by FAAS), assuming all the iron present in the sample exists as Fe_3_O_4_, and as it can be observed, the sample shows negligible coercive fields (Hc = 2.9 Oe) and remanence (M_R_ = 0.5 emu/g Fe_3_O_4_), corresponding to a nearly SPM NP behavior, characterized by an averaged zero magnetic moment in the absence of an externally applied magnetic field. In addition, the saturation magnetization of $M_{sat}^{NP}=62.8emu/g_{Fe3O4}$, below the bulk magnetite value 92 emu/gFe_3_O_4_, is consistent with a surface dead magnetic layer that lowers the total magnetization and is a characteristic signature of its small size [43] (average crystallite size, Dhkl, is ca. 10 nm, as estimated from the XRD using the Scherrer equation).

### 3.4. Transmission and Scanning Electron Microscopy (SEM, TEM, STEM, and EDS Mapping)

**Figure 4:**
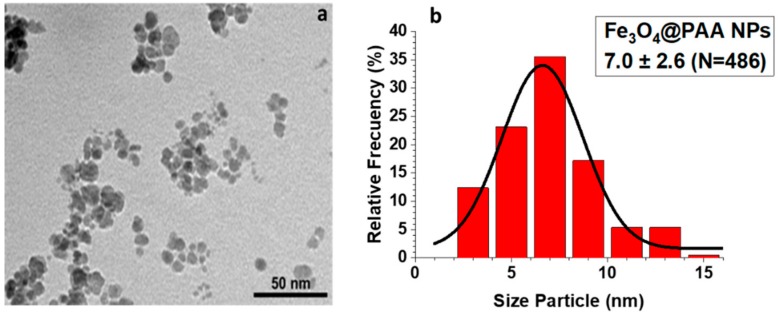
(**a**) Transmission electron microscopy (TEM) micrograph and (**b**) size distribution of the Fe_3_O_4_@PAA nanoparticles. The size distribution was performed by measuring a sample consisting of 486 magnetic nanoparticles with the ImageJ software.

In Figure 4a, a TEM micrograph of Fe_3_O_4_@PAA precursor is shown, which was obtained from a coprecipitation method, which was used to prepare the final Fe_3_O_4_@Al(OH)_3_ NPs. The magnetite cores have an irregular spherical morphology with a medium size distribution of around 7 nm (Figure 4b).

**Figure 5:**
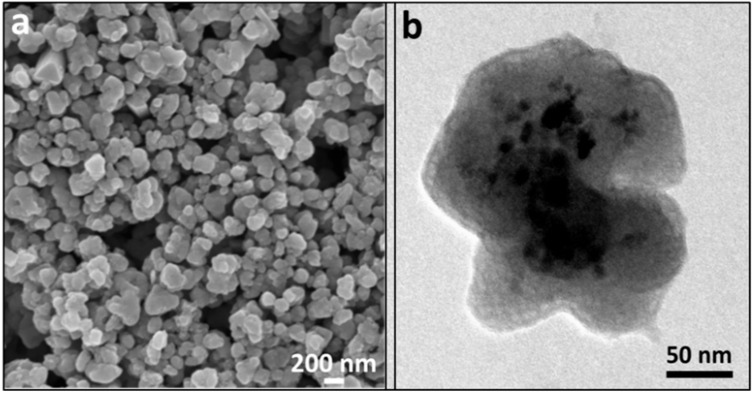
Scanning electron microscopy (SEM) (**a**) and TEM (**b**) micrographs of the Fe_3_O_4_@Al(OH)_3_ NPs.

SEM and TEM micrographs of Fe_3_O_4_@Al(OH)_3_ NPs (Figure 5) shows a nearly spherical morphology with a diameter of around 200–250 nm, where magnetite cores (dark contrast) are embedded inside the nanostructure. It can be appreciated in the TEM image (Figure 5b) that the aluminum hydroxide phase (light contrast) creates a wide and porous external layer, which helps in increasing the surface-to-volume ratio of the NP allowing to incorporate a higher amount of the desired radiolabeling payload resulting in great improvements in the detection capability. 

**Figure 6:**
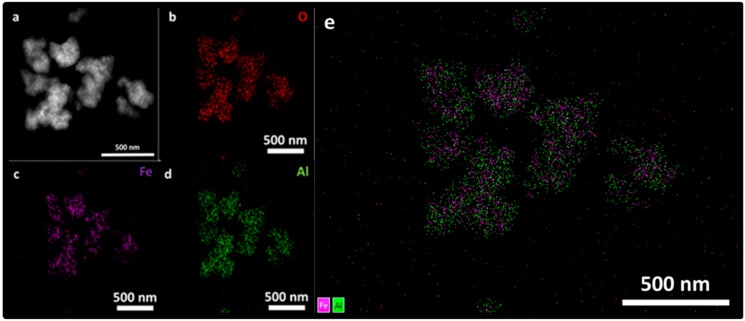
(**a**) STEM micrograph and mapping with energy dispersive X-ray (EDX) emitted by the elements present in the Fe_3_O_4_@Al(OH)_3_ samples: (**b**) oxygen, O (**c**) iron, Fe, (**d**) aluminum, Al, and (**e**) overlay of Fe (purple color) and Al (green colour) signal.

Additionally, a further assessment of the composition of the nanostructures was done by a STEM-energy dispersive X-ray spectroscopy (EDX) mapping and presented in Figure 6. STEM micrograph of the Fe_3_O_4_@Al(OH)_3_ NPs, is shown in Figure 6a and EDS mapping images of all the main constitutive elements present in the samples, oxygen, O, (Figure 6b), iron, Fe, (Figure 6c), and aluminum, Al, (Figure 6d) are homogeneously distributed. These results confirm the preliminary TEM assessment that aluminum hydroxide has adequately coated the magnetite cores, being the nanostructure available for both MRI/PET contrast activity.

### 3.5. Preliminary Study of Fluoride Adsorption of Non-Radioactive ^19^F^−^

Prior to the radiolabeling study of Fe_3_O_4_@Al(OH)_3_ NPs with ^18^F-sodium fluoride, a preliminary assay of non-radioactive fluoride adsorption (^19^F) was performed in order to test the adsorption capacity of the aluminum surface. It is well known that the solution’s pH of Al(OH)_3_ in a solvent has a decisive influence on the adsorption potential of the adsorbent since it alters the adsorbent surface charge [44,45]. It has been reported [46] that the fluoride ions adsorption ability of Fe_3_O_4_@Al(OH)_3_ NPs is highly increased when using acid pH solutions, for this reason, a mixture (described in Section 2.2.6), with pH 6.5 was selected for the present analysis. 

**Figure 7:**
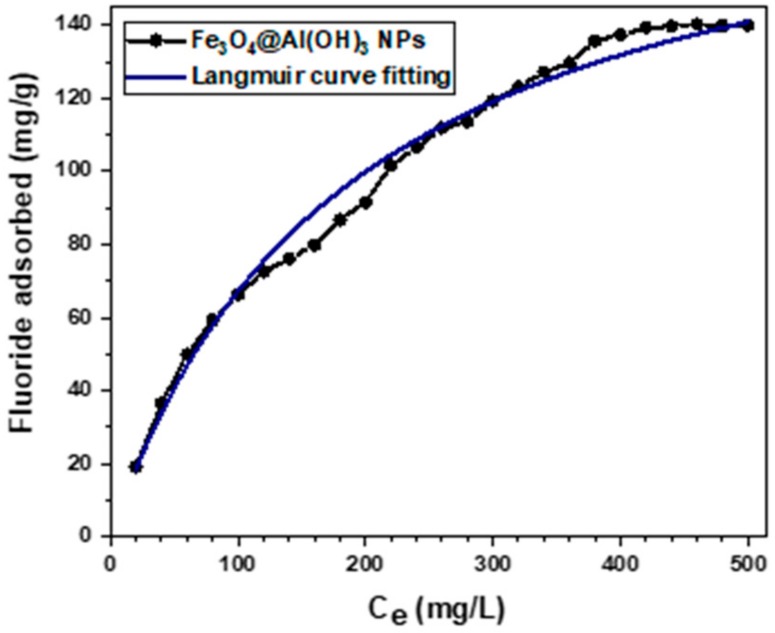
Equilibrium isotherm of fluoride adsorption by Fe_3_O_4_@Al(OH)_3_ NPs at 25 °C, pH 6.5.

In Figure 7, the equilibrium isotherm of fluoride adsorption is shown, for a set of 25 solutions with pH 6.5 and increasing concentrations of fluoride ions (Ce/mg L^−1^) and its fitting to a Langmuir adsorption model curve, which assumes a monolayer and homogeneous adsorption on a uniform adsorbent surface with energetically identical sorption sites where each molecules’ sorption energy is the same with no interaction between the adsorbed ones [47,48]. Langmuir adsorption is described by the following equation [49]:

(1) $$q_{e}=\frac{q_{m}K_{L}C_{e}}{1+K_{L}C_{e}}$$

where *q_e_* (mg g^−1^) is the mass of fluoride adsorbed per mass of adsorbent, *q_m_* (mg g^−1^) is the Langmuir constant correlated to the maximum capacity, *K_L_* (L mg^−1^) is the Langmuir constant related to the energy of adsorption and *C_e_* (mg L^−1^) is the concentration of fluoride at equilibrium.

The regression coefficient (R^2^) was 0.999; this high R^2^ shows a high fit with the Langmuir equation. The maximum adsorption capacity (*q_m_*) of fluoride was 194.03 mg/g Fe_3_O_4_@Al(OH)_3_ NPs, and the Langmuir adsorption constant (K_L_) was 0.005 L/mg. This *q_m_* value indicates the high affinity of this nanomaterial to adsorb fluoride as compared with previous work by Zhao et al. [46] and to be used for radiolabeling with ^18^F-fluoride. 

**Figure 8:**
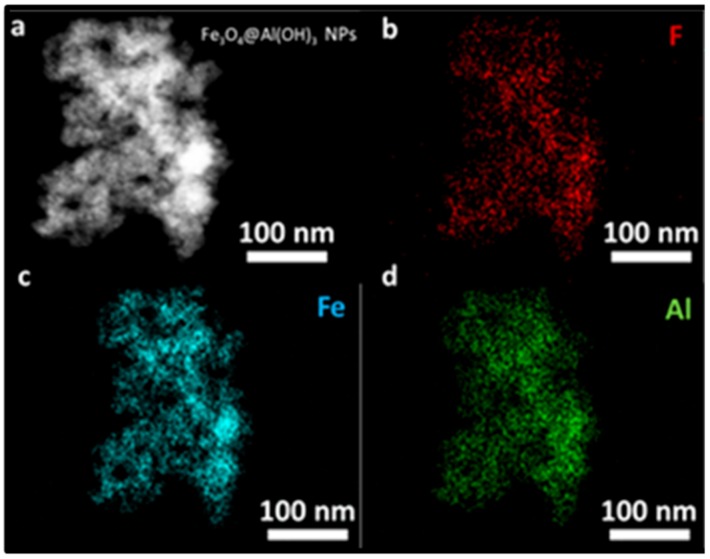
(**a**) STEM micrograph and mapping with EDX emitted by the elements present in the fluoride loaded Fe_3_O_4_@Al(OH)_3_ samples: (**b**) fluoride, F (**c**) iron, Fe, and (**d**) aluminum, Al.

In addition, STEM-EDX mapping was performed on the fluoride loaded samples to assess the distribution of the ions over the nanostructures. In Figure 8b, the presence of a high payload of fluoride is observed homogeneously distributed over the Al(OH)_3_ surface.

### 3.6. Time Course and Stability of [^18^F]NaF complexation to Fe_3_O_4_@Al(OH)_3_ NPs

**Figure 9:**
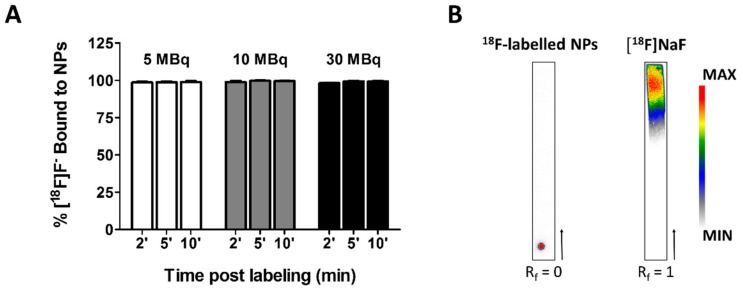
Time course of the radiolabeling of Fe_3_O_4_@Al(OH)_3_ NPs with [^18^F]F^−^. (**A**) Graphical representation of the percentage of [^18^F]F^−^ bound to NPs containing 1.34 mg/mL iron after two, five or ten minutes of labeling with 5, 10, or 30 MBq [^18^F]NaF. No significant differences were found between the different conditions (two-way repeated-measures ANOVA, Bonferroni correction), (**B**) Representative autoradiography of iTLC of (left) NPs loaded with [^18^F]F^−^ and (right) free [^18^F]NaF. The retardation factor (R_f_) of the labeled NPs indicates the complexation of the tracer to the NPs.

Next, NPs were labeled with 5–30 MBq radioactive [^18^F]NaF in Milli-Q water under constant shaking. Small samples volumes were collected for iTLC in eluting chambers containing 0.9% NaCl and subsequent analysis using either a gamma-counter or autoradiography (Figure 9).

Two minutes after the addition of 5 MBq of the radiotracer, 98.68% of the NPs were labeled with [^18^F]F^−^. The amount of bound [^18^F]F^−^ did not change significantly over time (98.72% and 98.94% after five and ten minutes, respectively; Figure 9A). Labeling the NPs with more radioactive ^18^F-fluoride (up to 30 MBq) did not affect the radiolabeling significantly (Figure 9A). Similar percentages of bound [^18^F]F^−^ were achieved. Performing autoradiography on the iTLC papers confirmed the results obtained by the gamma-counter as the retardation factor (R_f_) of the radiolabeled NPs is zero. In contrast, free [^18^F]NaF has an R_f_ of 1 (Figure 9B). Based on these results, we opted to radiolabel the NPs for ten minutes for all subsequent experiments.

As the radiolabeled NPs are meant for future cell and in vivo experiments, the stability of the adsorption of [^18^F]F^−^ to the NPs was investigated in different physiological media. Hereby, samples were collected after different times of exposure to the media and the [18F]F^−^ content in the NP was determined (Table 1). In a second experiment, NPs were resuspended in fresh media after each incubation and centrifugation steps (Supplementary Table S1). While the radiolabeling was relatively stable in Milli-Q water and saline, a steep decrease in the amount of [^18^F]F^−^ adsorbed to the NPs was seen already after 30 min after their submersion in PBS, cell culture medium for mMSCs, a mixture of 50% FBS, and 50% mMSCs medium or 100% FBS (Table 1). Most ^18^F release is associated with phosphate-containing media. Interestingly, loss of fluoride ions occurs within the first 30 min with only limited additional release during the following 4 h. Interestingly, repeated exposure to fresh media after each washing step and continued incubation did not result in similarly large drops in fluoride content (Supplementary Table S1).

### 3.7. PET/MRI Visualization of Fe_3_O_4_@Al(OH)_3_ NPs and NP-Loaded mMSC Labelled with [^18^F]NaF

**Figure 10:**
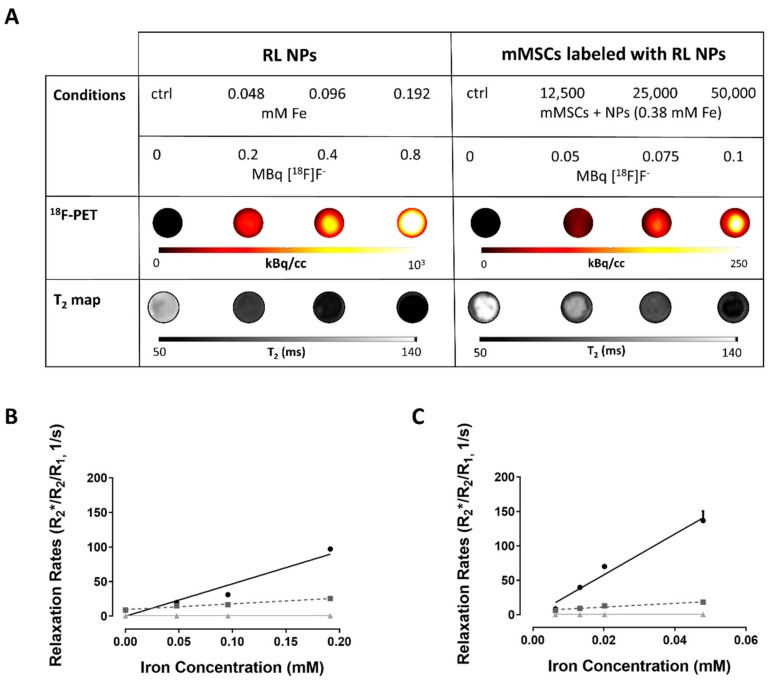
PET/MRI contrast properties of Fe_3_O_4_@Al(OH)_3_ NPs labeled with [^18^F]F^-^ and mouse mesenchymal stem cells (mMSCs) labeled with such NPs: (**A**) Upper row: The different conditions measured with the estimated radioactivity present at the start of measurements. Samples (100 µL) were diluted ½ with agar. Middle: ^18^F-positron emission tomography (PET) images and Bottom: T_2_ maps of the agar phantoms loaded with either radiolabeled Fe_3_O_4_@Al(OH)_3_ NPs (left) or mMSCs labeled with radiolabeled Fe_3_O_4_@Al(OH)_3_ NPs (right). (**B**,**C**) Relaxation rate (R_1_/_2_/_2_*) plotted against (**B**) iron concentration (mM) of Fe_3_O_4_@Al(OH)_3_ NPs or (**C**) intracellular iron concentration (mM) of mMSCs labeled with different amounts of radiolabeled Fe_3_O_4_@Al(OH)_3_ NPs. The following cell densities were studied: 12,500, 25,000, and 50,000 cells in 200 µL: 62,500 cells/mL, 125,000 cells/mL, and 250,000 cells/mL, respectively. The intracellular iron concentrations were obtained for 50,000 labeled cells.

The potential of the nanostructures as imaging and cell tracking agent was evaluated in a phantom, mimicking the conditions in the human body. Low amounts of radiolabeled NPs (0.048 mM iron, labeled with 2 MBq [^18^F]F^−^) and cells labeled with NPs (12,500 mMSCs containing 1.48 × 10^−3^ mM iron and labeled with approximately 0.05 MBq [^18^F]F^−^) could be visualized using simultaneously acquired PET/MRI (Figure 10A). 

The following relaxivities (r_x_) were obtained for the NPs: r_2_* = 468.8 ± 40.5 mM-1 s^−1^ (R^2^ = 0.93), r_2_ = 83.6 ± 4.5 mM^−1^ s^−1^ (R^2^ = 0.97), and r_1_ = NPs: 0.2 ± 0.04 mM^−1^ s^−1^ (R^2^ = 0.74). These were comparable or better than r_2_ values of other commercial contrast agents (Resovist) or experimental iron oxide NPs [23,50,51] (Figure 10B). Also, r_2_* was much higher compared to Resovist. As expected, T_2_/T_2_* values of labeled cells decreased with increasing cell numbers (Figure 10C). Even though the intracellular environment results in release of some ^18^F-fluoride from the NPs, the radiolabel was still suitable for mMSC visualization by PET. The count rates correlated with number of free NPs (iron content; R^2^ = 0.99) and number of cells, respectively (R^2^ = 0.98; Supplementary Figure S1).

In a preliminary proof-of-principle experiment, 10^5^ mMSCs labeled with RL NPs (10.51 pg ± 1.43 pg Fe per cell and 1.9 MBq [^18^F]F^−^) were injected intravenously in a wild-type C57Bl/6 mouse (see Supplementary Materials). We were able to visualize the uptake of the labeled cells/NPs in vivo, in the lungs and liver using PET/MRI, indicating the feasibility of this technique to detect the mMSCs (Supplementary Figure S2).

### 3.8. Assessment of Potentially Toxic Effects by Fe_3_O_4_@Al(OH)_3_ NPs

**Figure 11:**
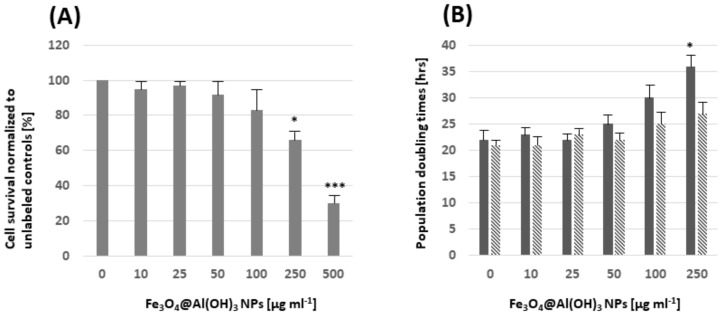
(**A**) Cell viability relative to the unlabeled control MSC was determined 12 h after exposure to Fe_3_O_4_@Al(OH)_3_ NPs post-labeling: A significant reduction in the cell survival was seen for concentrations > 100 μg NP mL^−1^. (**B**) Population doubling times (PDT) were determined after exposure of MSC to NPs for 12 h and subsequent incubation in NP-free medium for two days (solid bares) and six days (striped bares). Significant differences with respect to PDTs were only seen for concentrations of 250 μg NP mL^−1^ at two days after the removal of NPs from the medium. Three replicates were measured per condition. Differences relative to unlabeled cells were assessed based on a one-way ANOVA with Bonferroni correction. * *p* < 0.05 and *** *p* < 0.001.

Cell survival was assessed after a 12 h exposure of MSC to different concentrations of Fe_3_O_4_@Al(OH)_3_ NPs. No significant changes were noticed compared to the unlabeled cells for concentrations of up to 100 µg mL^−1^ NPs (Figure 11A). Cell proliferation was assessed at two and six days after the removal of NPs from the cell culture medium by determining population doubling times. Significant differences compared to unlabeled MSCs were only seen for 250 µg NPs mL^−1^ at two days after removal of the NPs from the medium (Figure 11B).

## 4. Discussion

We were able to demonstrate that Fe_3_O_4_@Al(OH)_3_ NPs are suitable contrast agents for PET and MR imaging. This provides the opportunity to overcome current limitations of PET (low resolution, limited half-life of radiotracers) and MRI (lower sensitivity, difficulty to visualize NPs in areas of low intrinsic signal intensity, for example lungs). This is in particular of interest for applications in cell tracking, like the monitoring of stem cell therapy, cell transplantation, or immunotherapeutic approaches [52,53,54,55,56,57].

In a first proof-of-concept study, we used Fe_3_O_4_@Al(OH)_3_ NPs for the labeling and tracking of mMSCs. As the working mechanism and the optimal route of administration of MSCs is still unknown and under debate [58], in vivo imaging and longitudinal tracking could help in understanding how they function under in vivo conditions. 

Characterization of our nanoparticles indicated high relaxivity even after cell labeling and engraftment of the cells in mice. In terms of adsorption of the PET tracer [^18^F]NaF, two minutes after the addition of 5 MBq of the radiotracer, 98.68% of the NPs were labeled with [^18^F]F^−^, which is comparable to previous work by Cui and colleagues [23,59].

The label remained stable in water. The addition of phosphates to the media clearly decreases the amount of [^18^F]F^−^ associated with the NPs starting from the initial measurement at 30 min. A possible explanation is the loss of the Al(OH)_3_ from the NPs and the competition between the fluoride and phosphate ions to bind to the Al(OH_)3_ shell of the NPs [23,59,60].

The steepest decrease at this early time point can be seen once the FBS medium is added. Even though the composition of the serum used is unknown, the potential presence of citrate, a known competitor for ^18^F-binding to Al(OH)_3_, could explain this very large drop in binding at early time points [8]. However, after the initial loss of fluoride ions from the NPs the loading remained stable over time, providing sufficient activity of the visualization of NPs and labeled cells. From the two possible explanations for the initial loss of fluoride ions from the NPs: (1) Adjustment to an equilibrium distribution bound/unbound [^18^F]F or (2) The maximum load of fluoride ions is exceeded by the initial load; the second explanation is supported by our data (Supplementary Figure S1), indicating that the initial ‘loss’ of radiolabel is most likely due to exposure to concentrations above loading capacity as the further ‘dilution’ of labeled nanoparticles results in only small additional loss of fluoride label.

The PAA coating of the NPs provided good biocompatibility in the concentration ranged used in our preliminary study. No indications of cell toxicity were seen for concentrations of up to 100 µg NP per mL of cell culture medium. 

Phantom experiments and cell labeling experiments focused on the visualization of mMSCs by using PET/MRI. Despite some in vitro loss of label, as shown in the stability tests, we were able to detect cells both in phantoms and in the mouse. In the preliminary mouse experiment, we chose to inject the RL NP-labelled cells intravenously as this method is also often used to study the mechanism of stem cells in cell therapy models [58,61,62]. The ability to visualize the labelled MSCs in vivo is encouraging for future biodistribution studies of disease models. Combining PET and MRI would open the opportunity for short-term follow-up and quantification of labelled cells with high specificity (PET) and long-term monitoring of the NP-labelled cells with high resolution (MRI) [63,64].

Utilization of [^18^F]F^−^ as a PET tracer has the disadvantage of a short half-life (110 min). But, compared to other tracers like ^89^Zr, advantages include a higher beta branching ratio, a higher volume sensitivity, and a lower partial volume effect, allowing better localization of the NPs [65,66].

Compared to the work of Cui et al. [23], the here presented nanomaterial shows a well-defined core/shell structure (see morphologic characterization, Figure 5) through controlled hydrolysis of aluminum hydroxide layer precursor salts ((Al(NO_3_)_3_·9H_2_O and Al_2_(SO_4_)_3_·16H_2_O). Our method to obtain hydrophilic Fe_3_O_4_@Al(OH)_3_ NPs provides advantages which are the employment of biocompatible and non-toxic starting reagents ((FeCl_3_·6H_2_O, FeSO_4_·7H_2_O, PAA and water as solvent)), efficient material production, highly reproducible and cost-effective procedures (synthesis reaction temperature <100 °C). Summarized, this can be understood as a great interest from the scientific and industrial point of view.

In contrast to the serial acquisition of PET and MR images [22], a simultaneous PET/MRI approach was applied in this study. This will provide not only spatial, but also temporal co-registration of PET and MR images. In addition, the acquisition time will be reduced [67,68]. These advantages allow future in vivo studies to focus on the validation of these NPs as cell tracking agents for longitudinal monitoring.

## 5. Conclusions

Efficient Fe_3_O_4_@Al(OH)_3_ NPs with controlled morphology were synthesized in situ by forced chemical hydrolysis method, characterized and applied as a novel contrast agent for MRI and PET imaging. Phantom studies of labeled NPs and mMSCs verified the high potential of these nanostructures to adsorb radioactive fluoride in aqueous solutions. In addition, these radiolabeled NPs and labeled mMSCs have excellent magnetic properties as superparamagnetic agents as well as good retention of [^18^F]F^−^ for visualization by MRI and PET, respectively. Furthermore, a simultaneous PET/MRI approach allowed the visualization of radiolabeled cells in a preliminary in vivo study. Further research will focus on the validation of these nanostructures as cell tracking agents.

## Figures and Tables

**Table 1:** Stability of [^18^F]F^−^ adsorption to Fe_3_O_4_@Al(OH)_3_ NPs after continued exposure to different media.

	[^18^F]F^−^ Bound to NPs after Storage at 37 °C for							
	30 min		1 h		2 h		4 h	
	*%*	*SD*	*%*	*SD*	*%*	*SD*	*%*	*SD*
Milli-Q water	65.6	17.2	85.6	5.2	77.9	6.3	86.7	8.3
Saline	69.5	2.6	66.8	12.9	78.5	12.7	76.9	12.4
PBS	57.4	5.6	53.8	12.7	38.5	2.1	40.1	5.0
TrypLE	53.1	5.7	61.1	19.6	38.2	5.1	36.9	3.4
mMSC medium	40.4	7.9	46.9	7.4	38.3	8.9	28.9	1.7
50% medium/ 50% FBS	36.8	3.3	58.5	14.8	40.3	3.2	40.4	5.2
FBS	35.7	1.8	46.5	8.1	32.5	3.3	34.4	10.3

Three replicates were measured per condition. No significant differences were found between the different time points based on a two-way repeated-measures ANOVA with Bonferroni correction for multiple comparisons.

## Supplementary Materials

The following are available online at https://www.mdpi.com/2079-4991/9/11/1626/s1, Supplementary Materials and Methods: In vivo PET/MRI of RL NPs-labelled mMSCs, Figure S1: Correlation of radiolabelled nanoparticles (RL NPs) and RL NP-labeled mouse mesenchymal stem cells (mMSCs) with radioactivity., Figure S2: In vivo positron emission tomography/magnetic resonance imaging (PET/MRI) images of a healthy mouse injected with mouse mesenchymal stem cells (mMSCs) labeled with ^18^F-labeled Fe_3_O_4_@Al(OH)_3_ NPs, Table S1: Stability of [^18^F]F^-^ adsorption to Fe_3_O_4_@Al(OH)_3_ nanoparticles (NPs) after repeated suspension of NPs in fresh media after each incubation and centrifugation step.

## Abbreviations

ATR	attenuated total reflectance
CT	computed tomography
DMEM	Dulbecco’s Modified Eagle’s Medium
ECA	elemental chemical analysis
EDS	energy dispersive X-ray spectroscopy
EDX	energy dispersive X-ray
FAAS	flame atomic absorption spectroscopy
FBS	fetal bovine serum
Fe_3_O_4_@OA	magnetite functionalized with oleic acid
FOV	field of view
FTIR	Fourier-Transform Infrared Spectroscopy
GOT	glutamate oxaloacetate transaminase enzyme
GPT	glutamate pyruvate transaminase enzyme
HMMSN	Hybrid Magnetic Mesoporous Silica Nanorods
HAADF	High-Angle Annular Dark Field
ICP-OES	inductively coupled plasma optical emission spectroscopy
ISE	Ion-Selective electrode
iTLC	instant thin layer chromatography
(m)MSCs	(mouse) mesenchymal stem cells
MNPs	magnetic nanoparticles
MRI	Magnetic Resonance Imaging
NPs	nanoparticles
PBS	Phosphate buffered saline
PDT	population doubling times
PET	Positron emission tomography
RL	radiolabelled
Rf	retardation factor
R_x_	relaxation rate
r_x_	relaxivity
SEM	scanning electron microscopy
SPECT	single photon emission computed tomography
SPM	superparamagnetic
STEM	scanning transmission electron microscopy
SUV	standardized uptake value
TGA	Thermogravimetric analysis
TEM	transmission electron microscopy
TISAB	III Total Ionic Strength Adjustment Buffer solution
VSM	vibrating sample magnetometer
XRD	X-ray diffraction.

## References

[1] Aiello M., Cavaliere C., Fiorenza D., Duggento A., Passamonti L., Toschi N. (2019). Neuroinflammation in neurodegenerative diseases: Current multi-modal imaging studies and future opportunities for hybrid PET/MRI. Neuroscience.

[2] Hang X., Xu K., Taratula O., Farsad K. (2019). Applications of nanoparticles in biomedical imaging. Nanoscale.

[3] Autio A., Uotila S., Kiugel M., Kytö V., Liljenbäck H., Kudomi N., Oikonen V., Metsälä O., Helin S., Knuuti J. (2019). ^68^Ga-DOTA chelate, a novel imaging agent for assessment of myocardial perfusion and infarction detection in a rodent model. J. Nucl. Cardiol..

[4] Garcia J., Tang T., Louie A. (2015). Nanoparticle-based multimodal PET/MRI probes. Nanomedicine.

[5] Eqqvist J. (2017). Nanoparticles as Theranostic Vehicles in Experimental and Clinical Applications-Focus on Prostate and Breast Cancer. Int. J. Mol. Sci..

[6] Li F., Zhi D., Luo Y., Zhang J., Nan X., Zhang Y., Zhou W., Qiu B., Wen L., Liang G. (2016). Core/shell Fe_3_O_4_/ Gd_2_O_3_ nanocubes as T1-T2 dual modal MRI contrast agents. Nanoscale.

[7] Kim D., Zhang Y., Kehr J., Klason T., Bjelke B., Muhammed M. (2001). Characterization and MRI study of surfactant-coated superparamagnetic nanoparticles administered into the rat brain. J. Magn. Magn. Mater..

[8] Jauregui-Osoro M., Williamson P., Glaria A., Sunassee K., Charoenphun P., Green M., Mullen G., Blower P. (2011). Biocompatible inorganic nanoparticles for [18F]–fluoride binding with applications in PET imaging. Dalt. Trans..

[9] Dadfar M.S., Roemhild K., Drude N., von Stillfried S., Knuechel R., Kiessling F., Lammers T. (2019). Iron oxide nanoparticles: Diagnostic, therapeutic and theranostic applications. Adv. Drug Deliv. Rev..

[10] Himmelreich U., Hoehn M. (2008). Stem cell labelling for Magnetic Resonance Imaging. Minimally Invasive Minim. Invasive Ther. Allied Technol..

[11] Liu S.F., Laurent H., Fattahi L.V.E., Muller R.N. (2011). Superparamagnetic nanosystems based on iron oxide nanoparticles for biomedical imaging. Nanomedicine.

[12] Piñeiro Y., Vargas Z., Rivas J., López-Quintela M.A. (2015). Iron Oxide based Nanoparticles for Magnetic Hyperthermia Strategies in Biomedical Applications. Eur. J. Inorg. Chem..

[13] Wang Y.X. (2011). Superparamagnetic iron oxide based MRI contrast agents: Current status of clinical application. Quant. Imaging Med. Surg..

[14] Laurent S., Boutry S., Mahieu I., Elst L.V., Muller R.N. (2009). Iron Oxide Based MR Contrast Agents: From Chemistry to Cell Labeling. Curr. Med. Chem..

[15] Sandiford L., Phinikaridou A., Protti A., Meszaros K.L., Cui X., Yan Y., Frodsham G., PWilliamson A., Gaddum N., Botnar M.R. (2012). Bisphosphonate-anchored PEGylation and radiolabeling of superparamagnetic iron oxide: Long-circulating nanoparticles for in vivo multimodal (T1 MRI-SPECT) imaging. ACS Nano.

[16] Berry C.C., Wells S., Charles S., Aitchison G., Curtis A.S.G. (2004). Cell response to dextran-derivatised iron oxide nanoparticles post internalisation. Biomaterials.

[17] Himmelreich U., Dresselaers T. (2009). Cell labelling and tracking for experimental models using Magnetic Resonance Imaging. Methods.

[18] Argibay B., Trekker J., Himmelreich U., Beiras A., Topete A., Taboada P., Perez-Mato M., Iglesias-Rey R., Sobrino T., Rivas J. (2016). Easy and efficient cell tagging with block copolymer-based contrast agents for sensitive MRI detection in vivo. Cell Transplant..

[19] Wang Y.X. (2015). Current status of superparamagnetic iron oxide contrast agents for liver magnetic resonance imaging. World J. Gastroenterol..

[20] The NanoTherm® Therapy. https://www.Magforce.Com/En/Home/Our_Therapy/.

[21] Kiani A., Esquevin A., Lepareur N., Bourguet P., Jeune F.L., Gauvrita J.Y. (2016). Main applications of hybrid PET-MRI contrast agents: A review. Contrast Media Mol. Imaging.

[22] Lee H.Y., Li Z., Chen K., Hsu A.R., Xu C., Xie J., Sun S., Chen X. (2008). PET/MRI dual-modality tumor imaging using arginine-glycine-aspartic (RGD)–conjugated radiolabelled iron oxide nanoparticles. J. Nucl. Med..

[23] Cui X., Belo S., Krüger D., Yan Y., de Rosales R.T.M., Jauregui-Osoro M., Ye H., Su S., Mathe D., Kovács N. (2014). Aluminum hydroxide stabilised MnFe2O4 and Fe3O4 nanoparticles as dual-modality contrasts agent for MRI and PET imaging. Biomaterials.

[24] Cui X., Green A.M., Blower J.P., Zhou D., Yan Y., Zhang W., Djanashvili K., Mathe D., Veres K.D.S. (2015). Szigeti. Chem. Commun..

[25] Wan Z., Chen W., Liu C., Liu Y., Dong C. (2015). Preparation and characterization of γ-AlOOH @CS magnetic nanoparticle as a novel adsorbent for removing fluoride from drinking water. J. Colloid Interface Sci..

[26] Chai L., Wang Y., Zhao N., Yang W., You X. (2013). Sulfate-doped Fe_3_O_4_/Al_2_O_3_ nanoparticles as a novel adsorbent for fluoride removal from drinking water. Water Res..

[27] Ayoob S., Gupta A.K. (2006). Fluoride in Drinking Water: A Review on the Status and Stress Effects. Crit. Rev. Environ. Sci. Technol..

[28] Zhang Y., Wang D., Liu B., Gao X., Xu W., Liang P., Xu Y. (2013). Adsorption of fluoride from aqueous solution using low-cost bentonite/chitosan beads. Am. J. Anal. Chem..

[29] Lodhia J., Mandarano G., Ferris N.J., Eu P., Cowell S.F. (2010). Development and use of iron oxide nanoparticles (part 1): Synthesis of iron oxide nanoparticles for MRI. Biomed. Imaging Interv. J..

[30] Xie L., Lan F., Li W., Liu Z., Ma S., Yang Q., Wu Y., Gu Z. (2014). Polyacrylic acid brushes grafted from P(St-AA)/Fe3O4 composite microspheres via ARGET-ATRP in aqueous solution for protein immobilization. Colloids Surf. B. Biointerfaces.

[31] Burugapalli K., Koul V., Dinda A.K. (2004). Effect of composition of interpenetrating polymer network hydrogels based on poly(acrylic acid) and gelatin on tissue response: A quantitative in vivo study. J. Biomed. Mater. Res..

[32] Massart R.M. (1981). Preparation of aqueous magnetic liquids in alkaline and acidic media. IEEE Trans. Magn..

[33] Roh H.-S., Choi G.-K., An J.-S., Cho C.-M., Kim D.-H., Park I.-J., Noh T.-H., Kim D.-W., Hong K.-S. (2011). Size-controlled synthesis of monodispersed mesoporous a-Alumina spheres by a template-free forced hydrolysis method. Dalton Trans..

[34] Trekker J., Leten C., Struys T., Lazenka V.V., Argibay B., Micholt L., Lambrichts I., Roy W.V., Lagae L., Himmelreich U. (2014). Sensitive in vivo cell detection using size-optimized superparamagnetic nanoparticles. Biomaterials.

[35] Gsell W., Molinos C., Correcher C., Junge S., Heidenberg M., Himmelreich U., Deroose C.M., Gonzalez J.G.A. (2019). Characterization of preclinical PET insert for 7T: Beyond NEMA testing. Proc. Intl. Soc. Mag. Reson. Med..

[36] Thevenaz P., Ruttiman U.E., Unser M. (1998). A pyramid approach to subpixel registration based on intensity. IEEE Trans. Image Process..

[37] Ketkar-Atre A., Struys T., Soenen J.S., Lambrichts I., Verfaillie C.M., de Cuyper M., Himmelreich U. (2013). Variability in contrast agent uptake by different but similar stem cell types. Intl. J. Nanomed..

[38] Kimura S., Ming L.C., Manghnani H.M., Nakagiri N. (1986). Physics and Chemistry of Minerals.

[39] O’Daniel H., Zigan F., Rothbauer R. (1967). Zeitschrift fuer Kristallographie, Kristallgeometrie, Kristallphysik. Kristallchemie.

[40] Fu P., Lu W., Lei W., Wu K., Xu Y., Wu J. (2013). Thermal Stability and Microstructure Characterization of MgAl2O4 Nanoparticles Synthesized by Reverse Microemulsion Method. Mater. Res..

[41] Wang Y.L., Bao J., Wang L., Zhang F., Li Y.D. (2006). One-Pot Synthesis and Bioapplication of Amine-Functionalized Magnetite Nanoparticles and Hollow Nanospheres. Chem. Eur. J..

[42] Liao M.H., Chen D.H. (2002). Preparation and characterization of a novel magnetic nano-adsorbent. J. Mater. Chem..

[43] Goya G.F., Berquo T.S., Fonseca F.C. (2003). Static and dynamic magnetic properties of spherical magnetite nanoparticles. J. Appl. Phys..

[44] Amalraj A., Pius A. (2017). Removal of fluoride from drinking water using aluminum hydroxide coated activated carbon prepared from bark of Morinda tinctorial. Appl. Water Sci..

[45] Rosenqvist J., Persson P., Sjöberg S. (2002). Protonation and charging of nanosized gibbsite (α-Al(OH)3) particles in aqueous suspension. Langmuir.

[46] Zhao X., Wanga J., Wub F., Wanga T., Caia Y., Shia Y., Jianga G. (2010). Removal of fluoride from aqueous media by Fe3O4@Al(OH)3 magnetic nanoparticles. J. Hazard. Mater..

[47] Bitton G. (1998). Formula Handbook for Environmental Engineers and Scientist.

[48] Kumar V.K., Sivanesan S. (2006). Selection of optimum sorption kinetics: Comparison of linear and non-linear method. J. Hazard. Mater..

[49] Langmuir I. (1916). The Constitution and Fundamental Properties of Solids and Liquids. Part I. Solids. J. Am. Chem. Soc..

[50] Klug G., Kampf T., Bloemer S., Bremicker J., Ziener C.H., Andrea H., Bureck U.G., Rommel E., Nöth U., Schenk W.A. (2010). Intracellular and extracellular T1 an T2 relaxivities of magneto-optical nanoparticles at experimental high fields. Magn. Reson. Med..

[51] Richard S., Boucher M., Lalatonne Y., Mériaux S., Motte M. (2017). Iron oxide nanoparticle surface decorated with cRGD peptides for magnetic resonance imaging of brain tumors. Biochim. Biophys. Acta Gen. Subj..

[52] Aarntzen E.H.J.G., Figdor C.G., Adema G.J., Punt C.J.A., de Vries I.J.M. (2008). Dendritic cell vaccination and immune monitoring. Cancer Immunol. Immunother.

[53] Jirak D.J.K., Berkova Z., Herynek V., Lodererova A., Girman P., Habart D., Hajek M., Saudek F. (2012). Detection of pancreatic islet allograft impairment in advance of functional failure using magnetic resonance imaging. Transpl. Int..

[54] Malosio M.L., Esposito A., Brigatti C., Palmisano A., Piemonti L., Nano R., Maffi P., de Cobelli A.F., Maschio D., Secchi A. (2015). MR imaging monitoring of iron labelled pancreatic islets in a small series of patients: Islets fate in successful, unsuccessful and auto-transplantation. Cell Transpl..

[55] De Chickera S., Willert C., Mallet C., Foley R., Foster P., Dekaban G.A. (2011). Cellular MRI as a suitable, sensitive non-invasive modality for correlating in vivo migratory efficiencies of different dendritic cell populations with subsequent immunological outcomes. Int. Immunol..

[56] De Temmerman M.-L., Soenen S.J., Symens N., Lucas B., Vandenbroucke R.E., Libert C., Demeester J., de Smedt S.C., Himmelreich U., Rejman J.R. (2014). Magnetic layer-by-layer coated particles for efficient magnetic resonance imaging of dendritic cells and mesenchymal stem cells. Nanomedicine.

[57] Argibay B., Trekker J., Himmelreich U., Beiras A., Topete A., Taboada P., Pérez-Mato M., Vieites-Prado A., Iglesias-Rey R., Rivas J. (2017). Intraarterial route increases the risk of cerebral lesions after mesenchymal cell administration in animal model of ischemia. Sci. Rep..

[58] Eggenhofer E., Benseler V., Kroemer A., Popp F.C., Geissler E.K., Schlitt H.J., Baan C.C., Dahlke M.H., Hoogduijn M.J. (2012). Mesenchymal stem cells are short-lived and do not migrate beyond the lungs after intravenous infusion. Front. Immunol..

[59] McBride W.J., Sharkey R.M., Goldenberg D.M. (2013). Radiofluorination using aluminum-fluoride (Al18F). EJNMMI Res..

[60] Cleeren F., Lecina J., Billaud E.M.F., Ahamed M., Verbruggen A., Bormans G.M. (2016). New Chelators for Low Temperature Al18 F-Labelling of Biomolecules. Bioconjug. Chem..

[61] Schrepfer S., Deuse T., Reichenspurner H., Fischbein M.P., Robbins R.C., Pelletier M.P. (2007). Stem Cell Transplantation: The Lung Barrier. Transplant. Proc..

[62] Fischer U.M., Harting M.T., Jimenez F., Monzon-Posadas W.O., Xue H., Savitz S.I., Laine G.A., Cox C.S. (2009). Pulmonary passage is a major obstacle for intravenous stem cell delivery: The pulmonary first-pass effect. Stem Cells Dev..

[63] Massoud T.F., Gambhir S.S. (2003). Molecular imaging in living subjects: Seeing fundamental biological processes in a new light. Genes Dev..

[64] Kiessling F.K. (2008). Noninvasive Cell Tracking. Molecular Imaging II.

[65] Conti M., Eriksson L. (2016). Physics of pure and non-pure positron emitters for PET: A review and a discussion. EJNMMI Phys..

[66] De Jong H.W.A.M., Perk L., Visser G.W.M., Boellaard R., van Dongen G.A.M.S., Lammertsma A.A. High Resolution PET Imaging Characteristics of ^68^Ga, ^124^I and ^89^Zr Compared to ^18^F. Proceedings of the IEEE Nuclear Science Symposium Conference Record, 2005.

[67] Lahooti A., Sarkar S., Laurent S., Shanehsazzadeh S. (2016). Dual nano-sized contrast agents in PET/MRI: A systematic review. Contrast Media Mol. Imaging..

[68] Mannheim J.G., Schmid A.M., Schwenck J., Katiyar P., Herfert K., Pichler B.J., Disselhorst J.A. (2018). PET/MRI Hybrid Systems. Semin. Nucl. Med..

