# Supplementary material for: Development of Superparamagnetic Nanoparticles Coated with Polyacrylic Acid and Aluminum Hydroxide as an Efficient Contrast Agent for Multimodal Imaging
Source: Nanomaterials (Basel). 2019 Nov 15;9(11):1626. doi: 10.3390/nano9111626 (PMC6915788; doi:10.3390/nano9111626)
Supplement: Supplementary file 1 [file nanomaterials-09-01626-s001.pdf]

Supplementary Materials: Development of Superparamagnetic Nanoparticles Coated with Polyacrylic Acid and Aluminum Hydroxide as an Efficient Contrast Agent for Multimodal Imaging

Manuel Antonio González-Gómez [1,*,†], Sarah Belderbos [2,3,*,†], Susana Yañez-Vilar [1], Yolanda Piñeiro [1], Frederik Cleeren [4], Guy Bormans [4], Christophe M. Deroose [3,5], Willy Gsell [2,3], Uwe Himmelreich [2,3] and Jose Rivas [1]

[1] Applied Physics Department, NANOMAG Laboratory, Universidade de Santiago de Compostela, 15782 Santiago de Compostela, Spain; susana.yanez@usc.es (S.Y.-V.); y.pineiro.redondo@usc.es (Y.P.); jose.rivas@usc.es (J.R.)

[2] Biomedical MRI, Department of Imaging and Pathology, KU Leuven, O&N I, Herestraat 49—Box 505, 3000 Leuven, Belgium; willy.gsell@kuleuven.be (W.G.); uwe.himmelreich@kuleuven.be (U.H.)

[3] Molecular Small Animal Imaging Center (MoSAIC), KU Leuven, O&N I, Herestraat 49—Box 505, 3000 Leuven, Belgium

[4] Radiopharmaceutical Research, Department of Pharmaceutical and Pharmacological Sciences, KU Leuven, O&NII Herestraat 49—Box 821, 3000 Leuven, Belgium; frederik.cleeren@kuleuven.be (F.C.); guy.bormans@kuleuven.be (G.B.)

[5] Nuclear Medicine and Molecular Imaging, Department of Imaging and Pathology, KU Leuven/UZ Leuven, Herestraat 49—Box 7003 59, 3000 Leuven, Belgium; christophe.deroose@uzleuven.be

* Correspondence: manuelantonio.gonzalez@usc.es (M.A.G.-G.); sarah.belderbos@kuleuven.be (S.B.)

† The authors contributed equally to this work.

Supplementary Materials and Methods

In vivo PET/MRI of RL NPs-labelled mMSCs

The in vivo experiment was approved by the Ethical Committee of the KU Leuven (ECD n° 259/2015). It was conducted according to the Belgian (Royal Decree of 29 May 2013), Flemish (Decision of the Flemish Government to adapt the Royal Decree of 29 May 2013, 17 February 2017), and European (Directive 2010/63/EU) regulations on the protection of animals used for scientific purposes. Mice were housed in individually ventilated cages and had access to food and water *ad libitum*. In vivo procedures described were performed under anaesthesia (2% isoflurane in 100% O_2, IsoVET, 100 mg/g, Eurovet, Piramal Healthcare, London, UK).

A total of 3 * 10^5 cells were plated overnight and were labelled the next day with saline containing NPs (0.38 mM iron; radiolabelled with 10 MBq [^{18}F]NaF). 1*10^5 radiolabelled cells (0.19 MBq upon injection; 10.51 ± 1.43 pg Fe per cell) were injected in a healthy seven-week-old wild-type C57Bl/6 mice via the tail vein, and their biodistribution was studied using simultaneous PET/MRI as described above.

A static one-hour PET scan was acquired simultaneously with a 3D T_2-weighted anatomical MRI scan (RARE sequence with effective TE = 26 ms, TR = 500 ms, rare factor = 8, matrix = 150 × 350 × 128, FOV = 35 mm × 80 mm × 30 mm, acquisition time = 13 min 30 s) and a respiration-gated T_2 map of the liver (multi slice multi echo sequence with TE = 8 – 64 ms with 8 ms increments, TR = 1800 ms, 2 averages, matrix = 192 × 128, FOV = 42 mm × 30 mm, two intercalated slice packages with each eight slices of 1 mm and a 1 mm slice gap, acquisition time = approximately 14 min).

PET images and PET/MRI overlays were created as described in the main text. Standardized uptake value (SUV) normalization of the PET images was performed according to the following formula:

$$SUV = \frac{[Activity]_{\text{organ}}}{[injected\ activity/weight\ of\ animal]}$$

Supplementary Figures and Tables

Table S1. Stability of [^{18}F]F$^-$ adsorption to $Fe_3O_4@Al(OH)_3$ nanoparticles (NPs) after repeated suspension of NPs in fresh media after each incubation and centrifugation step.

	**[^{18}F]F$^-$ bound to NPs after different centrifugation steps (20' at 4,000 rpm)**							
	**Step 1**		**Step 2**		**Step 3**		**Step 4**	
	%	***SD***	***%***	***SD***	***%***	***SD***	***%***	***SD***
Milli-Q water	96.4	0.5	97.3	0.5	95.3	2.8	97.6	1.0
mMSC medium	91.5	5.5	70.4 **	11.6	80.2	4.6	76.2 *	16.4
50% medium/ 50% FBS	89.1	3.6	79.0	10.4	75.6	7.1	75.8	5.7
FBS	92.1	0.4	79.5	6.8	81.3	2.5	79.6	2.4

Three replicates were measured per condition. In each cycle, nanoparticles (NPs) were resuspended in fresh media, followed by 15-minute exposure and a centrifugation step (20 minutes at 4,000 rpm). Indicated statistics are based on a mixed-effect analysis with Bonferroni correction for multiple comparisons and are all in comparison to step 1. * $p < 0.05$, ** $p < 0.01$.

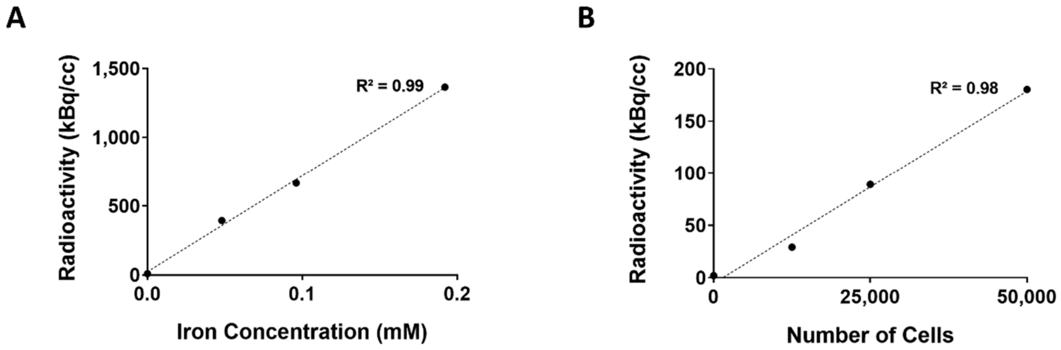

Figure S1: Correlation of radiolabelled nanoparticles (RL NPs) and RL NP-labeled mouse mesenchymal stem cells (mMSCs) with radioactivity. A good correlation was found between **A**) the iron content (mM) present in samples containing radiolabeled (RL) NPs or **B**) the number of cells present in samples containing RL NP-labeled mMSCs and the amount of radioactivity present in the respective tubes (kBq/cc; Pearson correlation, $p < 0.01$ for both conditions).

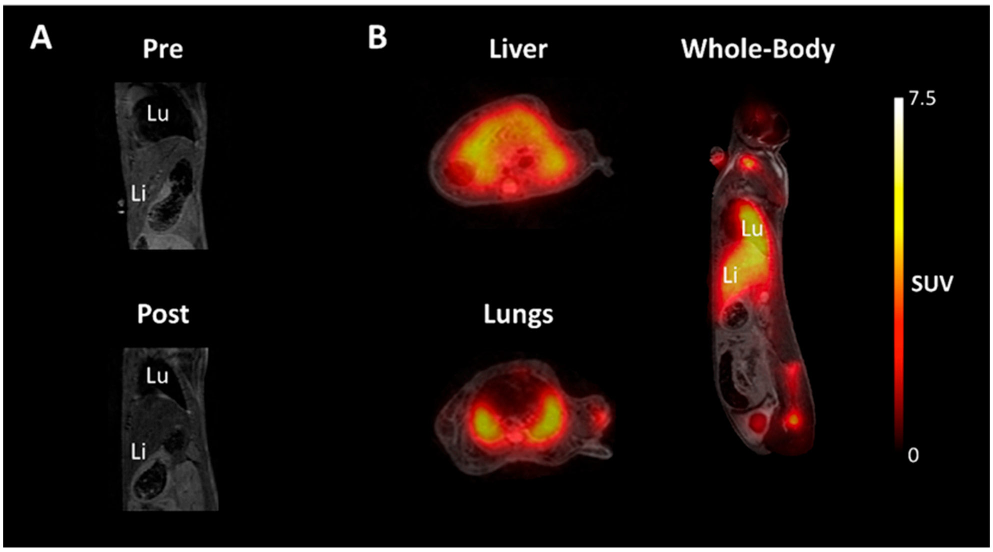

Figure S2: In vivo positron emission tomography/magnetic resonance imaging (PET/MRI) images of a healthy mouse injected with mouse mesenchymal stem cells (mMSCs) labeled with ^{18}F-labeled $Fe_3O_4@Al(OH)_3$ NPs. **A**) Sagittal views of 3D T_2-weighted MRI scans pre and post-injection of the labeled mMSCs. Cell uptake in the lungs (Lu) cannot be visualized, while a slight darkening of the liver (Li) indicates the presence of NPs. **B**) Overlays of 3D T_2-weighted MRI images and 1 h static PET images show the presence of the labeled cells/ nanoparticles in the lungs and in the liver on both the axial panels (left) and the sagittal panel (right). Units = standardized uptake values (SUV).
